# Evaluation of the effectiveness of comprehensive drug price reform: a case study from Shihezi city in Western China

**DOI:** 10.1186/s12939-020-01246-9

**Published:** 2020-08-06

**Authors:** Taoyu Lin, Zhaohui Wu, Menming Liu, Xiangwei Wu, Xinping Zhang

**Affiliations:** 1 School of Medicine and Health Management, Tongji Medical College, Huazhong University of Science and Technology, Wuhan, 430030 China; 2 The First Affiliated Hospital, School of Medicine, Shihezi University, Xinjiang, 832008 China; 3 Social Insurance Administration Bureau in Shihezi City, Xinjiang, 832008 China

**Keywords:** Comprehensive drug price reform, Medical expense, Medical service utilisation, Reimbursement, Basic medical insurance, Interrupted time-series

## Abstract

**Background:**

China carried out a comprehensive drug price reform (CDPR) in 2017 to control the growing expense of drug effectively and reduce the financial burden of inpatients. However, early studies in pilot regions found the heterogeneity in the effectiveness of CDPR from different regions and other negative effects. This study aimed to evaluate the effects of the reform on medical expenses, medical service utilisation and government financial reimbursement for inpatients in economically weaker regions.

**Methods:**

Shihezi was selected as the sample city, and 238,620 inpatients, who were covered by basic medical insurance (BMI) and had complete information from September 2016 to August 2018 in public hospitals, were extracted by cluster sampling. An interrupted series design was used to compare the changing trends in medical expenses, medical service utilisation and reimbursement of BMI for inpatients before and after the reform.

**Results:**

Compared with the baseline trends before the CDPR, those after the CDPR were observed with decreased per capita hospitalisation expenses (HE) by ¥301.9 per month (*p* < 0.001), decreased drug expense (DE) ratio at a rate of 0.32% per month (*p* < 0.05) and increased ratio of diagnosis and treatment expenses (DTE) at a rate of 0.25% per month (*p* < 0.01). The number of inpatients in secondary and tertiary hospitals declined by 458 (*p* < 0.001) and 257 (*p* < 0.05) per month, respectively. The BMI reimbursement in tertiary hospitals decreased by ¥254.7 per month (*p* < 0.001).

**Conclusion:**

The CDPR controlled the increase in medical expenses effectively and adjusted its structure reasonably. However, it also reduced the medical service utilisation of inpatients in secondary and tertiary hospitals and financial reimbursement for inpatients in tertiary hospitals.

## Introduction

The rapid increase in drug price puts considerable strain on the affordability and sustainability of healthcare spending [1, 2]. According to a report on global drug use survey, global drug expenditures that reached $1.2 trillion in 2018 will exceed $1.5 trillion by 2023, and the average annual growth rate will be 3 to 6%. Meanwhile, most (73%) of the global drug expenditure is in US, EU5 (i.e. Germany, France, Italy, U.K. and Spain), Japan and China [3]. In the past decades, many countries have explored the implementation paths of drug price reforms, such as drug–price regulation, payment reform of medical insurance, combination of drug–price regulation and drug–use reduction, to curb the rising drug expenditures [2, 4–8]. These reforms have reduced drug prices in the short-term but are unsustainable and have even produced unexpected results, such as a rebound in drug costs [4, 7–9].

In China, the long-term surge of drug expense (DE) is because medical service prices are lower than the labour costs of medical staff [10]. However, public hospitals can make a 15% profit in prescribing drugs to make up for the deficit in the low prices of medical services [11]. The relationship between hospital medical revenue and DE has led to an artificial rise in drug prices and a surge in hospitalisation expenses (HE) [12, 13]. Another reason for the increase in DE is associated with the basic medical insurance (BMI) payment regime. In China, the BMI fund compensates inpatients for medical expenses more than outpatients [14]. Moreover, the compensations for medical expenses amongst inpatients vary by hospitals. That is, the higher the hospital tier, the lower the compensations of medical expenses. The purpose is to reduce the financial burden of inpatients and guide the patients to rationally choose a hospital according to their health status [15]. However, it has not limited the patients’ behaviour for seeking healthcare service [16], so a large number of patients seeking high-quality medical service bypass primary hospitals to visit secondary and tertiary hospitals, thereby increasing unnecessary medical expenses [17]. According to statistics, the DE in public hospitals exceeded 30% of the per capita HE between the years 2007 and 2017, up to even 40% (Additional file 1 Fig. S1), far higher than 17% in the member countries of the Organization for Economic Co-operation and Development [7]. In the face of this situation, the Chinese government promulgated three guiding opinions on the pilot of the comprehensive reform of urban public hospitals, the reform of medical service price and the reform of BMI payment methods from 2015 to 2017 [15, 18, 19]. The comprehensive drug price reform (CDPR) has begun to take shape.

Shihezi, a city of Xinjiang Production and Construction Corps (XPCC), is located in western China where the economic development lags behind the eastern and central regions [20]. Meanwhile, it is one of the core region of China’s “silk road economic belt” and the maintenance of national stability. At present, the city’s population accounts for 21% of the XPCC’s total population, and its health expenditure exceeds 3% of the national average health expenditure [21, 22]. According to statistics, the population aged 65 years and older in Shihezi accounts for 17% of the total population in 2015, exceeding the national average of 5.5%, and the prevalence of chronic diseases has reached 599.3‰ [23, 24]. These findings can further aggravate the utilisation rate of hospitalisation services and stimulate the growth of health expenses [25]. In 2017, all public hospitals in China have implemented the CDPR officially [26]. In September 2017, Shihezi launched the CDPR, which involves three aspects, namely, eliminating the 15% drug profit, increasing the medical service price to make up the loss of drug price and scientifically adjusting BMI payment standards to ensure that the first two reforms do not increase the financial burden of patients.

Literature review revealed that DE shows a downward trend in regions where the above reforms have started early. By the end of 2016, more than 90% of public hospitals from 200 pilot cities nationwide have cancelled drug markup, and the proportion of DE in HE have shown a negative growth (from 36.8% in 2015 to 34.6% in 2016) for the first time [27]. However, the accumulated evidences in the literature showed that HE paid by the BMI funds also has a downward trend [28–30]. A study found that the drug price level in western low-income regions such as Gansu and Yunnan was higher than that in eastern high-income regions such as Beijing and Jiangsu, suggesting that the CDPR may ignore the imbalance of economic development in various regions [31]. A study from economically developed region found that some public hospitals made more profits by over-delivery of medical services and over-admission of patients [32]. Above evidences indicated differences in the actual implementation of reform policy by local governments at the local level, the lack of effective linkage between the policies of adjusting medical service price and adjusting BMI payment, and the excessive utilisation of medical services. So far, except for a study on a single-institution [33], no large population size has been used to study the effectiveness of the CDPR in the XPCC. Based on the above situation, it is urgent to clarify the effects of the CDPR in this city, which will have an important impact on the population health and economic development of XPCC in the future.

In view of the positive and negative effects of early drug price reforms in the pilot regions and the heterogeneity in the effects of CDPR from different regions, the objective of the current study is to evaluate the effectiveness of CDPR through the changing trends in medical expenses, medical service utilisation and government financial reimbursement for inpatients before and after the CDPR in Shihezi. These findings will provide the basis for promoting the health equity of the residents in regions with weak economic development and provide a reference for countries and regions with similar of economical development and health expenditure to formulate and improve drug reform policies.

## Materials and methods

### Data sources

Data were obtained from the database of the Social Insurance Administration Bureau in Shihezi City. Cluster sampling was used to extract the data of 245,730 inpatients covered by BMI in public hospitals between September 2016 and August 2018 from the database. The collected data were processed anonymously, and each patient was identified by a unique identification code. Excluding 7110 inpatients with incomplete data information, 238,620 inpatients from 30 public hospitals were retained.

The data included baseline characteristics and medical expenses for inpatients. The baseline characteristics included age (< 65 and ≥ 65), sex (male and female), medical institution (primary, secondary and tertiary hospitals) and BMI [urban employee medical insurance (UEMI) and urban and rural resident medical insurance (URRMI)]. The medical expenses for inpatients included HE, DE, diagnosis and treatment expense (DTE) and BMI reimbursement.

### Study design

An interrupted time series design was used to compare the per capita medical expenses of inpatients before and after the CDPR that came into effect on September 1, 2017 [34]. The interrupted time series analysis (ITSA) is the strongest quasi experimental study that can effectively evaluate the change in the outcome rate in the time periods before and after implementation of a policy, especially in the absence of a control group [35]. In the present study, the effect of policy changes was measured using the following equation:

$$ {Y}_t={\beta}_0+{\beta}_1\times time+{\beta}_2\times pol\mathrm{i} cy+{\beta}_3\times time afterpolicy+{\varepsilon}_t $$

In the equation, *Y*_*t*_ is the independent outcome variable of medical expenses at time *t* (i.e. time unit is the number of months). *Time* is a continuous variable representing the months since the start of the study, *policy* is a dummy (indicator) variable representing the pre-CDPR (September 2016 to August 2017 and coded 0) and the post-CDPR (September 2017 to August 2018 and coded 1) periods and *time after policy* is a continuous variable representing the number of months since September 2017 and is coded as zero before September 2017. *β*_0_ represents the baseline level of the outcome variable, *β*_1_ represents the slope of the outcome variable before the reform, *β*_2_ represents the change in the outcome level occurring in the period immediately following the implementation of the reform and *β*_3_ represents the difference between pre- and post-reform slopes of the outcome [36].

### Outcome variables

Eight outcome variables, namely, number of inpatients, per capita HE, per capita DE and DE ratio, per capita DTE and DTE ratio, per capita BMI reimbursement and reimbursement ratio, were analysed (Table 1). The number of inpatients was measured as an indicator of medical service utilisation in inpatients. The per capita HE and DE and DTE ratios were measured as indicators to evaluate the effects of the control and structural adjustment for medical expenses. The BMI reimbursement and the reimbursement ratio were measured as indicators to reflect government financial reimbursement for inpatients.

**Table 1:** Outcome variables and calculation formulas

Outcome variables	Calculation formula
Number of inpatients	The sum of the number of inpatients over a period of time.
Per capita HE	The sum of HE for all inpatients over a period of time/the number of inpatients in the same period.
Per capita DE	The sum of DE for inpatients over a period of time/the number of inpatients in the same period.
DE ratio	Per capita DE over a period of time/per capita HE in the same period × 100%.
Per capita DTE	The sum of DTE for all inpatients over a period of time/the number of inpatients in the same period.
DTE ratio	Per capita DTE over a period of time/per capita HE in the same period × 100%.
Per capita BMI reimbursement	The sum of BMI reimbursement for all inpatients over a period of time/the number of inpatients in the same period.
Reimbursement ratio	Per capita BMI reimbursement over a period of time/per capita HE in the same period × 100%.

*Abbreviation*: *BMI* basic medical insurance, *DE* drug expense, *DTE* diagnosis and treatment expense, *HE* hospitalization expense;

### Statistical analysis

Data analysis consisted of three stages. Firstly, the baseline characteristics between the pre- and the post-CDPR periods were analysed as frequencies and percentages for categorical variables. Secondly, the outcome variables were calculated monthly between September 2016 and August 2018. Finally, ITSA was used to analyse the effects of implementing the CDPR. The Newey–West method was used to handle heteroscedasticity and autocorrelation. The Actest was used to test autocorrelation and determine the number of time-series lags to ensure that the model conforms to the correct autocorrelation structure [36]. The STATA statistical software version 14 was used for this analysis.

## Results

### Inpatient characteristics and medical expenses

The final sample included 238,620 inpatients. A total of 123,980 and 114,640 were included in the pre- and post-CDPR periods, respectively. The age, sex and medical insurance distributions of inpatients were similar between the two CDPR periods (Table 2). The median age was 61 years (interquartile range = 49 years to 74 years), 43.8% of total inpatients were 65 years and older, 54.5% were female, and 77.9% were UEMI. The distribution of inpatients admitted to primary hospitals increased from 43.0 to 50.9%, and the distributions of inpatients in secondary and tertiary hospitals decreased by 4.5 and 3.3%, respectively (Table 2).

**Table 2:** Inpatients characteristics and medical expenses

Indicators	Overall (*N* = 238,620)	Pre-CDPR period (*n* = 123,980)	Post-CDPR period (*n* = 114,640)
Age (years)			
Median (IQR)	61.0 (49–74)	60.0 (48–74)	62.0 (49–75)
≥ 65	104,580 (43.8)	52,956 (42.7)	51,624 (45.0)
< 65	134,040 (56.2)	71,024 (57.3)	63,016 (55.0)
Gender			
Female	130,061 (54.5)	67,461 (54.4)	62,600 (54.6)
Male	108,559 (45.5)	56,519 (45.6)	52,040 (45.4)
Medical Institution			
Primary hospital	111,658 (46.8)	53,331 (43.0)	58,327 (50.9)
Secondary hospital	50,955 (21.4)	29,192 (23.5)	21,763 (19.0)
Tertiary hospital	76,007 (31.9)	41,457 (33.4)	34,550 (30.1)
BMI			
UEMI	185,774 (77.9)	96,630 (77.9)	89,144 (77.8)
URRMI	52,846 (22.1)	27,350 (22.1)	25,496 (22.2)

Data are n (%). *Abbreviation*: *CDPR* comprehensive drug price reform, *IQR* interquartile range, *UEMI* urban employee medical insurance, *URRMI* urban and rural resident medical insurance

### ITSA results of medical expenses and its compositions

**Fig. 1:**
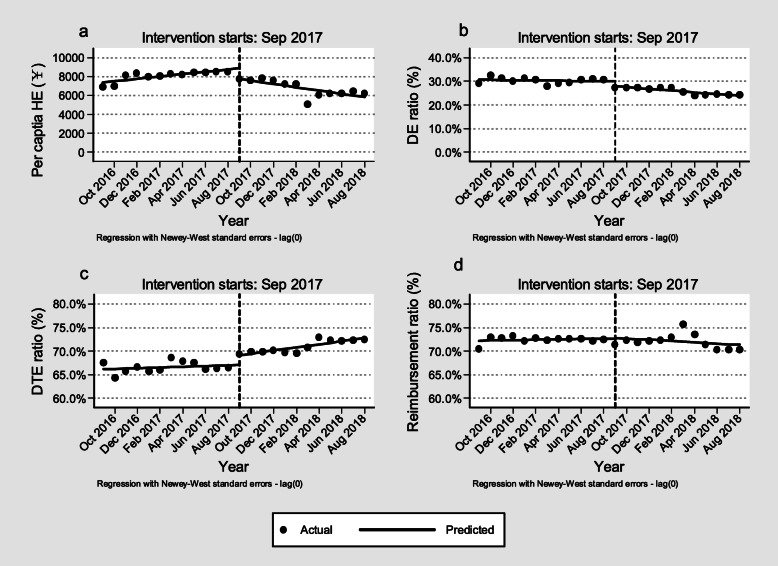
The per capita HE ant its composition in the pre- and post-CDPR periods. Note: **a** Per capita HE. **b** DE ratio. **c** DTE ratio. **d** Reimbursement ratio. Abbreviation: CDPR, comprehensive drug price reform. DE, drug expense. DTE, diagnosis and treatment expense. HE, hospitalization expense. RMB, renminbi, Chinese currency

In the pre-CDPR period, evident growth trends were observed in per capita HE (¥124.2 per month; 95% *CI* = 52.8 to 195.6, *p* < 0.01), and the DE and DTE ratios remained unchanged. However, the introduction of the CDPR was followed by sudden reductions in per capita HE (¥ − 1112.4; 95% *CI* = − 1610.5 to − 614.3, *p* < 0.001) and DE ratio (− 2.08% per month; 95% *CI* = − 3.52 to − 0.64%, *p* < 0.01) and a significant increase in DTE ratio (2.04%; 95% *CI* = 0.71 to 3.36, *p* < 0.01). Compared with the baseline trends in the pre-CDPR period, the per capita HE in the post-CDPR period decreased by ¥301.9 per month (95% *CI* = − 394.8 to − 208.9, *p* < 0.001), DE ratio decreased at a rate of 0.32% per month (95% *CI*:− 0.55 to − 0.08%, *p* < 0.05) and the DTE ratio increased at a rate of 0.25% per month (95% *CI* = 0.04 to 0.46%, *p* < 0.01). In the entire study period, no statistically significant difference in imbursement ratio was observed (Fig. 1). The ITSA results of ITSA of each outcome variable were reported in additional file 2 Table S1 and S2.

### ITSA results of medical utilisation in different hospitals

**Fig. 2:**
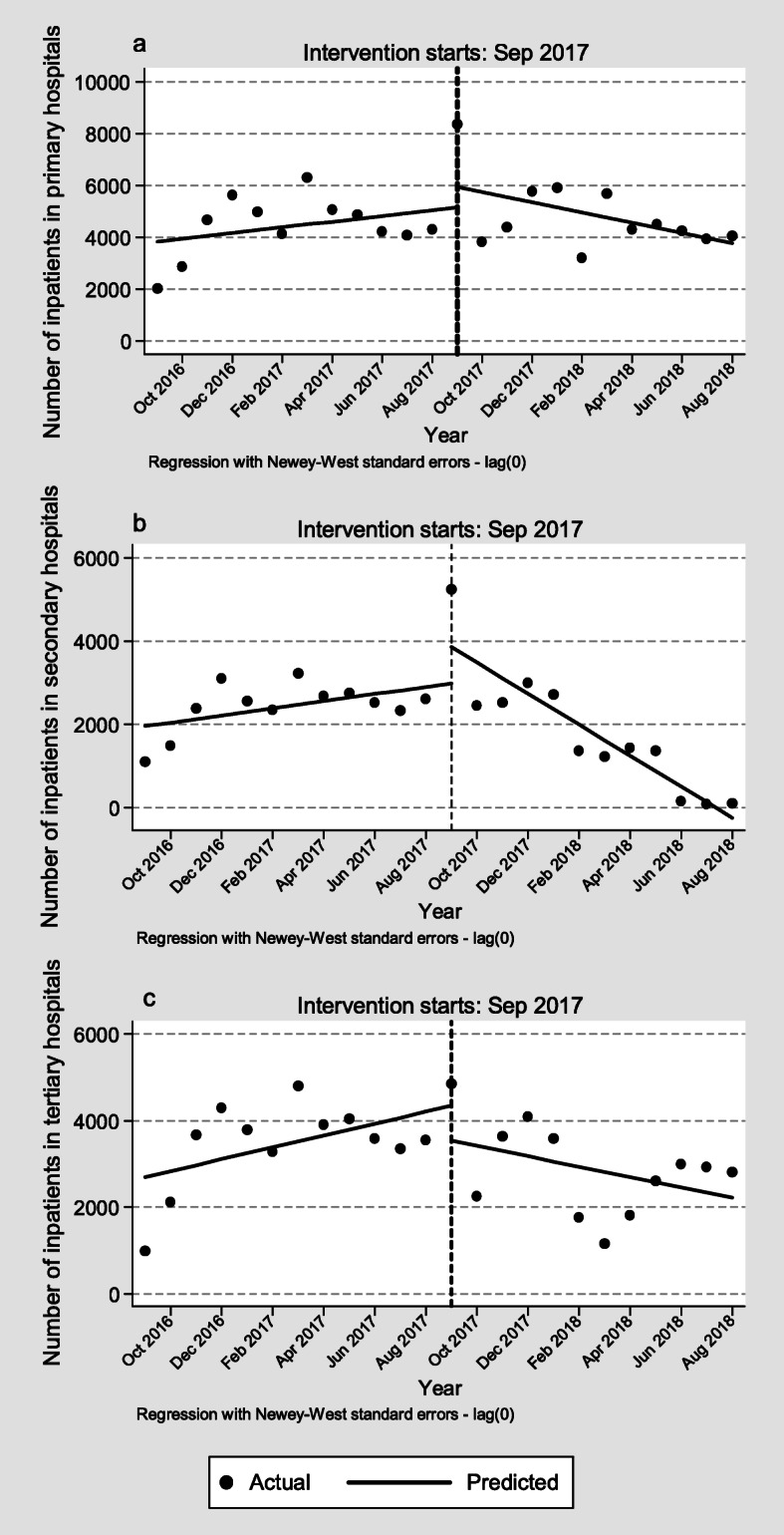
The number of inpatients in the pre- and post-CDPR periods. Note: **a** Number of inpatients in primary hospitals. **b** Number of inpatients in secondary hospitals. **c** Number of inpatients in tertiary hospitals. Abbreviation: CDPR, comprehensive drug price reform

In the pre-CDPR period and the start of the CDPR, no statistically significant difference was found in the number of inpatients in different hospitals. However, compared with the difference between the pre- and post-CDPR slopes, significant reductions were found in the number of inpatients in secondary (− 458 per month, 95% *CI* = − 643 to − 272, *p* < 0.001) and tertiary (− 257 per month, 95% *CI* = − 510 to − 4, *p* < 0.05) hospitals. No significant difference was found in the number of inpatients in primary hospitals (Fig. 2). The ITSA results of medical utilisation in different hospitals were reported in additional file 2 Table S3.

### ITSA results of government financial reimbursement in different hospitals

**Fig. 3:**
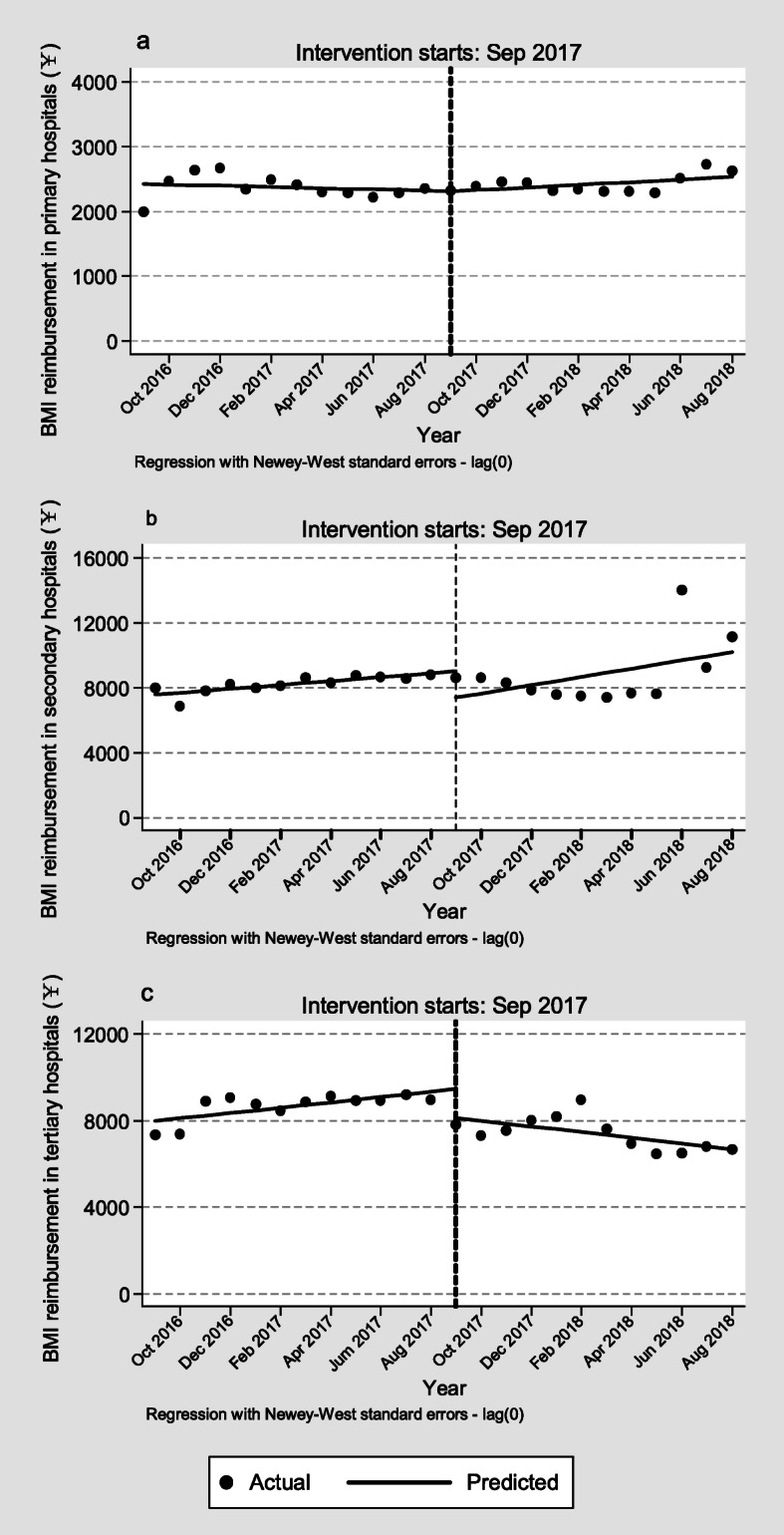
The BMI reimbursement in the pre- and post-CDPR periods. Note: **a** BMI reimbursement in primary hospitals. **b** BMI reimbursement in secondary hospitals. **c** BMI reimbursement in tertiary hospitals. Abbreviation: BMI, basic medical insurance. CDPR, comprehensive drug price reform

During the entire study period, the per capita BMI reimbursement in primary hospitals remained stable and had no significant change. The per capita BMI reimbursement in secondary hospitals presented a trend of growth (¥120.3 per month, 95% *CI* = 48.1 to 192.5, *p* < 0.01) in the pre-CDPR period and sharply declined (¥ − 1613.4 per month, 95% *CI* = − 2926.6 to − 300.1, *p* < 0.05) in the start of the CDPR implementation, but the change in the pre- and post-CDPR slopes had no significant difference (*p* = 0.378). The per capita BMI reimbursement in tertiary hospitals had an increase of ¥122.8 per month (95% *CI* = 24.8 to 220.8, *p* < 0.05) in the pre-CDPR period and a sharp decline (¥ − 1327.9 per month; 95% *CI* = − 2115.2 to − 540.5, *p* < 0.01) in the period immediately following reform. Moreover, the change in the trends of the pre- and post-CDPR slopes had a statistically significant decrease (¥ − 254.7 per month, 95% *CI* = − 376.0 to − 133.5, *p* < 0.001) (Fig. 3). The ITSA results of BMI reimbursement in different hospitals were reported in additional file 1 Table S4.

## Discussion

Since the CDPR, our study shows that the per capita HE presents an expected downward trend, in which the DE share gradually decreased and the DTE share increased accordingly. Moreover, the overall utilisation of medical service was a decrease of 9340 person-times compared with that before the reform. The government’s financial reimbursement for inpatients has changed.

Our finding corroborated previous research showing the CDPR implementation effectively controls the rapid growth of HE and adjusts its structure [29, 37, 38]. These previous studies focused more on the impact of CDPR on medical expenses than on BMI reimbursement or medical service utilisation, or both. Through in-depth analysis of the changing trend of BMI reimbursement and medical service utilisation in different hospitals, we found that the decrease of medical service utilisation was mainly focused on that in large hospitals such as secondary and tertiary hospitals, and the main change of government financial reimbursement was a decline in the BMI reimbursement level for inpatients in tertiary hospitals. Among the few studies that comprehensively analyzed the impacts of CDPR on HE, BMI reimbursement and medical service utilization, a study [32] from high-income region found that the CDPR increased the BMI reimbursement level, thus stimulating inpatients to over-utilize medical service in large hospitals. Another study [28] from a region with similar economic development to this study found that the CDPR had raised the BMI reimbursement level in tertiary hospitals. The above studies used descriptive methods to analyze the changing trend of BMI reimbursement and medical service utilisation, which could not exclude the possibility of an upward or downward trend before the intervention. Therefore, our study complements the evidence on the impact of CDPR on the BMI reimbursement and medical service utilisation.

A reduction of HE could stimulate more patients to release the needs for hospitalization services. This study found that after the reform, the utilisation of medical services not only didn’t increase, but reduced utilisation of medical service in secondary and tertiary hospitals didn’t trigger an influx of patients into primary hospitals. Under the influence of many factors such as economic development and medical resources, the medical service utilisation in western China has been lower than that in other regions [39]. This finding implies a further deterioration in the low utilisation of medical service in this region. The result should be related to the BMI payment. In addition to adjusting reimbursement, the reform of BMI payment launched the global-budget payment system and payment by the type of disease as part of the CDPR. These two payment methods, by setting each hospital’s annual budget and the payment criteria for each common disease, and adopting the principle of surplus retention and non-compensation of overspending, stimulated each hospital to actively control the increase in the overall and personal medical expenses and reduce inappropriate admission [40–42]. However, when deciding on admission, doctors may prefer to choose patients with no comorbidities, complications or unserious illness to avoid exceeding the annual budget apportioned to the department and the payment limit for the diseases [43]. Therefore, the reduction of inpatients should include both those who are improperly admitted and those with sevious illness who need to be admitted but are refused. This finding shows that the implementation of the CDPR reduces the medical service utilisation of patients with severe diseases and lessens the chances of their access to timely and effective treatment.

The decrease of BMI reimbursement level in tertiary hospitals after the CDPR demonstrates that Shihezi government has reduced the compensation for patients with critical and severe diseases. The medical service function in tertiary hospitals in China is to rescue, diagnose and treat patients with critical and severe illness [44]. Thus, the condition of inpatients who meet the admission standards of tertiary hospitals should be more serious than that in other hospitals. BMI reimbursement is designed to provide financial risk protection for patients against poverty caused by disease [45]. This finding demonstrates that after the CDPR, the out-of-pocket medical expenses of inpatients in tertiary hospitals are increasing. From the patients’ perspective, this not only increases the financial burden on inpatients with relatively severe and complex diseases, but may also be a key factor that affects access to medical services for patients with poverty who meet the criteria for hospital stay in tertiary hospitals.

There are several limitations observed in this study. A major limitation is that the accessibility of data limits comparative analysis with other representative cities to evaluate the effectiveness of the CDPR more comprehensively. A long-term multiregional evaluation is needed to evaluate the overall effect of the reform objectively and comprehensively. A report on the similarities and differences between the effects and objectives of CDPR in a city from Western China is provided as a basis for future studies. The difference in the severity of inpatients’ illness before and after the reform cannot be determined due to the limited data. Thus, the influence of this confounding factor on HE cannot be ruled out. At present, the payment model of disease-related groups for inpatients’ care is still in the pilot stage [46]. For further studies, the combination index of case in the payment method can be used to reflect the severity of the disease, the complexity of diagnosis and treatment methods, the amount of resource consumption, and evaluate the effectiveness of the reform more scientifically and objectively. Finally, the models in this study assume a linear trend of outcome variables before and after the intervention. In fact, the possibility of non-linear trends also exists. Although some models, such as Box-Jenkins models, may be used to analyze non-linear trends, they are less useful in examining changes in trend occurring at defined time points [47]. In this study, 12 data points before and after the CDPR and more than 100 observations per data point were collected, which can provide the robust estimates of change in level and trend for outcomes [48].

Although the study has limitations, our findings provide important implications for the health development of CDPR in regions with weak economic development. The reduction of medical service utilisation and the diminution of financial compensation for patients with critical and severe implicates that the reform of the medical insurance payment system in the CDPR only focuses on the control of medical expenses and the adjustment of the structure of DE and DTE, but ignores the correct guidance of medical service utilisation and the financial risk protection of patient. It illustrates that synchronising the suite of policies is affected by the fragmented governance of medical care, medicine and medical insurance. Therefore, when formulating the specific measures of the CDPR, local governments need to adapt to the local conditions and give full play to the incentive and compensation mechanisms of medical insurance, rather than mechanical control medical expenses and restructuring them.

## Conclusions

Overall, the CDPR has decreased the per capita HE and per capita DE amongst the inpatients of Shihezi significantly and has a positive role in increasing DTE. Results demonstrate that the CDPR controls the increase in medical expenses and reasonably adjusts its structure. However, the CDPR reduces the medical service utilisation of inpatients in secondary and tertiary hospitals and sharply declines the government financial reimbursement for inpatients in tertiary hospitals. The former may affect the timely and effective treatment for patients with serious diseases, whereas the latter increases the financial burden on inpatients from tertiary hospitals and may hinder access to medical services for patients who meet the criteria for admission to tertiary hospitals. These findings will affect the health equity of population in this aging city, eventually affecting the process of reform.

## Supplementary information

**Additional file 1: Fig. S1.** The proportion of per capita DE in per capita HE in China, 2007–2017.

**Additional file 2 : Table S1.** ITSA results of medical expenses. **Table S2.** ITSA results of the distribution of medical expense. **Table S3.** ITSA results of medical utilisation in different hospitals. **Table S4.** ITSA results of BMI reimbursement in different hospitals.

## Data Availability

The datasets generated and/or analyzed during the current study are not publicly available due to the data ownership belongs to the Social Insurance Administration Bureau in Shihezi City. Interested parties could contact the Social Insurance Administration Bureau in Shihezi City to request access to data.

## Abbreviations

BMI: basic medical insurance
CDPR: comprehensive drug price reform;
DE: drug expense
DTE: diagnosis and treatment expense
HE: hospitalization expense
ITSA: interrupted time series analysis
RMB: renminbi
UEMI: urban employee medical insurance
URRMI: urban and rural resident medical insurance
XPCC: Xinjiang Production and Construction Corps.
