# Supplementary material for: Evaluation of the effectiveness of comprehensive drug price reform: a case study from Shihezi city in Western China
Source: Int J Equity Health. 2020 Aug 6;19:133. doi: 10.1186/s12939-020-01246-9 (PMC7409685; doi:10.1186/s12939-020-01246-9)
Supplement: Supplementary file 1 — Additional file 1: Fig. S1. The proportion of per capita DE in per capita HE in China, 2007–2017. [file 12939_2020_1246_MOESM1_ESM.docx]

**Additional file 1**

There is 1 figure in additional file 1.

 **Fig. S1** The proportion of per capita DE in per capita HE in China, 2007-2017.

The data come from China Health Statistics Yearbook. Abbreviation: HE, hospitalization expense. DE, drug expense.
