# Supplementary material for: Evaluation of the effectiveness of comprehensive drug price reform: a case study from Shihezi city in Western China
Source: Int J Equity Health. 2020 Aug 6;19:133. doi: 10.1186/s12939-020-01246-9 (PMC7409685; doi:10.1186/s12939-020-01246-9)
Supplement: Supplementary file 2 — Additional file 2 : Table S1. ITSA results of medical expenses. Table S2. ITSA results of the distribution of medical expense. Table S3. ITSA results of medical utilisation in different hospitals. Table S4. ITSA results of BMI reimbursement in different hospitals. [file 12939_2020_1246_MOESM2_ESM.docx]

**Additional file 2**

There are 4 tables in additional file 2.

**Table S1** ITSA results of medical expenses

| Outcome variable | Pre-intervention trend | Level change | Trend change |
| --- | --- | --- | --- |
| Per capita HE, ¥ | 124.2^**^  (52.8 to 195.6) | −1112.4^***^  (−1610.5 to −614.3) | −301.9^***^  (−394.8 to −208.9) |
| Per capita DE, ¥ | 33.8^*^  (7.9 to 59.7) | −508.6^***^  (−676.3 to −340.9) | −105.5^***^  (−137.3 to −73.6) |
| Per capita DTE, ¥ | 88.7^**^  (38.0 to 139.4) | −576.7^**^  (−946.2 to −207.2) | −192.0^***^  (−256.8 to −127.3) |
| Per capita BMI reimbusement, ¥ | 92.2^**^  (33.0 to 151.3) | −805.2^***^  (−1170.0 to −440.5) | −228.8^***^  (−298.1 to −159.4) |

Notes: Data are coefficient, the numbers in parentheses indicate 95% confidence interval. ^*^ *P* < 0.05 ^**^ *P* < 0.01 ^***^ *P* < 0.001

**Table S2** ITSA results of the distribution of medical expense

| Outcome variable | Pre−intervention trend | Level change | Trend change |
| --- | --- | --- | --- |
| DE ratio, % | −0.05  (−2.8 to 0.2) | −2.08^**^  (−3.52 to −0.64) | −0.32^*^  (−0.55 to −0.08) |
| DTE ratio, % | 0.08  (−0.12 to 0.28) | 2.04^**^  (0.71 to 3.36) | 0.25 ^**^  (0.04 to 0.46) |
| Reimbursement ratio, % | 0.04  (−0.13 to 0.21) | 0.12  (−1.34 to 1.56) | −0.17  (−0.43 to 0.09) |

Notes: Data are coefficient, the numbers in parentheses indicate 95% confidence interval. ^*^ *P* < 0.05 ^**^ *P* < 0.01 ^***^ *P* < 0.001

**Table S3** ITSA results of medical utilisation in different hospitals

| Outcome variable | Pre−intervention trend | Level change | Trend change |
| --- | --- | --- | --- |
| Number of inpatients in primary hospitals | 109.5  (−102 to 321.4) | 778.3  (−1685.5 to 3242.0) | −304.8  (−644.2 to 34.6) |
| Number of inpatients in secondary hospitals | 85.8  (−17.8 to 189.4) | 869.2  (−443.6 to 2182.0) | −457.8^***^  (−642.7 to −272.8) |
| Number of inpatients in tertiary hospitals | 137.0  (−51.8 to 325.9) | −806.3  (−2461.6 to 848.9) | −257.0^*^  (−510.2 to −3.9) |

Notes: Data are coefficient, the numbers in parentheses indicate 95% confidence interval. ^*^ *P* < 0.05 ^**^ *P* < 0.01 ^***^ *P* < 0.001

**Table S4** ITSA results of BMI reimbursement in different hospitals

| Outcome variable | Pre−intervention trend | Level change | Trend change |
| --- | --- | --- | --- |
| Primary hospitals | −9.4  (−52.2 to 33.5) | −2.8  (−218.3 to −212.7) | 29.6  (−18.0 to 77.2) |
| Secondary hospitals | 120.3^**^  (48.1 to 192.5) | −1613.4^*^  (−2926.6 to −300.1) | 134.9  (−176.9 to 446.6) |
| Tertiary hospitals | 122.8^*^  (24.8 to 220.8) | −1327.9^**^  (−2115.2 to −540.5) | −254.7^***^  (−376.0 to −133.5) |

Notes: Data are coefficient, the numbers in parentheses indicate 95% confidence interval. ^*^ *P* < 0.05 ^**^ *P* < 0.01 ^***^ *P* < 0.001
